# **Tobacco** ‘**antisense’ lines with a stepwise reduction in Rubisco allowed a network approach to the regulation of photosynthesis, metabolism, allocation and growth**

**DOI:** 10.1007/s00425-026-04928-w

**Published:** 2026-02-04

**Authors:** Mark Stitt

**Affiliations:** Max Planck Institute of Molecular Plant Physiology, Am Muehlenberg 1, 14476 Potsdam-Golm, Germany

**Keywords:** Antisense, Calvin–Benson cycle, Gene expression, Flux control coefficient, Network analysis, Photosynthesis, Rubisco

## Abstract

**Main conclusion:**

Over 2 decades ago, antisense *rbcS *tobacco lines with a progressive decrease in Rubisco abundance allowed network analysis of the regulation of photosynthesis, metabolism, and whole plant allocation.

**Abstract:**

In the 1970 and 1980s, the study of the regulation of metabolism and growth was largely descriptive. Conceptual frameworks had been formulated that would allow a more rigorous approach, for example, to generate a small decrease in enzyme abundance and measure the resulting change in pathway flux. The lack of suitable mutants, however, made this approach practically impossible. This changed drastically when *Agrobacterium*-mediated transformation made it possible to alter expression of enzymes and other proteins at will. (Quick et al. in Planta 183:542–554, 1991a) and subsequent papers used antisense lines with a progressive decrease in Rubisco abundance to show that the contribution of Rubisco to the control of photosynthesis varies greatly, depending on the conditions in which photosynthesis is occurring and the conditions in which the plants had been grown. Analogous experiments by us and others on other Calvin–Benson cycle enzymes showed that they could also exert control and that the distribution of control depended on the conditions. We also used the *rbcS* antisense lines to, first, show that small decrease in Rubisco abundance is often accommodated by the photosynthetic apparatus to minimize the inhibition of photosynthesis and, second, explore how a larger decrease in Rubisco abundance and the resulting inhibition of photosynthesis impacts on central carbon and nitrogen metabolism, specialized metabolism, and whole plant architecture. This approach anticipated future developments like network analysis and system biology, is still relevant to designing strategies to improve crop photosynthesis, and can provide insights into photosynthetic performance and trade-offs in the field in a fluctuating environment.

## How metabolic regulation was investigated in the 1970 and 1980s

The setting is the end of the 1980 s, when the first transgenic plants with altered expression of enzymes were being generated. Until then, experimental studies of regulation involved comparisons along a developmental series or between conditions. Gene technology revolutionized the research of metabolism and physiology; it opened up the possibility of altering the expression of a chosen gene and looking at the consequences. This transformed plant physiology into molecular plant physiology, and laid the foundation for the later emergence of systems biology. In Quick et al. (1991a) and a series of following publications, this emerging technology was used to dissect the contribution of Rubisco to the control of photosynthesis, to ask how other reactions in photosynthesis respond to a decrease in Rubisco abundance, and to probe the relationship between photosynthesis, plant architecture, and growth.

The approach used in the 1970 and 1980 s to study the regulation of metabolism was nicely laid out by Newsholme and Start (1973). The first step was to identify enzymes that catalyze non-equilibrium or ‘irreversible’ reaction. This involved measuring in vivo metabolite levels and calculating the free energy change of each reaction in the pathway. Enzymes that catalyze non-equilibrium or ‘irreversible’ reactions were highlighted for further studies. Enzymes that catalyze near-equilibrium or ‘reversible’ reactions and therefore catalyze both, the forward and reverse reactions, at high rates were assumed to be present in large excess, and hence of little or no importance for regulation. The next step was to compare metabolite levels along a developmental sequence or in conditions in which flux changed, and ask which metabolite(s) change in the opposite direction to pathway flux. If pathway flux rises and a metabolite falls, the implication is that the enzyme that uses this metabolite has been activated. An analogous logic applies in cases where flux fell; here the experimenter would search for an enzyme whose substrate rose. This allowed one or more ‘regulatory’ enzymes to be short-listed. The next step was to discover the mechanism. The enzyme would be characterized in vitro to discover metabolites that activate or inhibit it, or post-translational regulation mechanisms (the latter was exceedingly challenging back then, and is still not trivial!). This information would be used to find metabolites whose in vivo response could explain the inferred in vivo activation or inhibition of the ‘regulatory’ enzyme. This approach was laborious and time-consuming. It was also often difficult to apply, for example, at metabolic branchpoints (if a metabolite drops, which of the 2 enzymes is responsible!) and when there was subcellular compartmentation (as is typically the case in plants!). Information from studies that applied this approach is still an invaluable resource, but it was often difficult to identify conclusively which enzyme(s) regulate(s) metabolism in one condition, let alone across a range of conditions and organs. It was also not possible to decide which was ‘the most important’—to quantify contributions of different enzymes. A useful critique is provided by Fell and Thomas (1995).

**Fig. 1:**
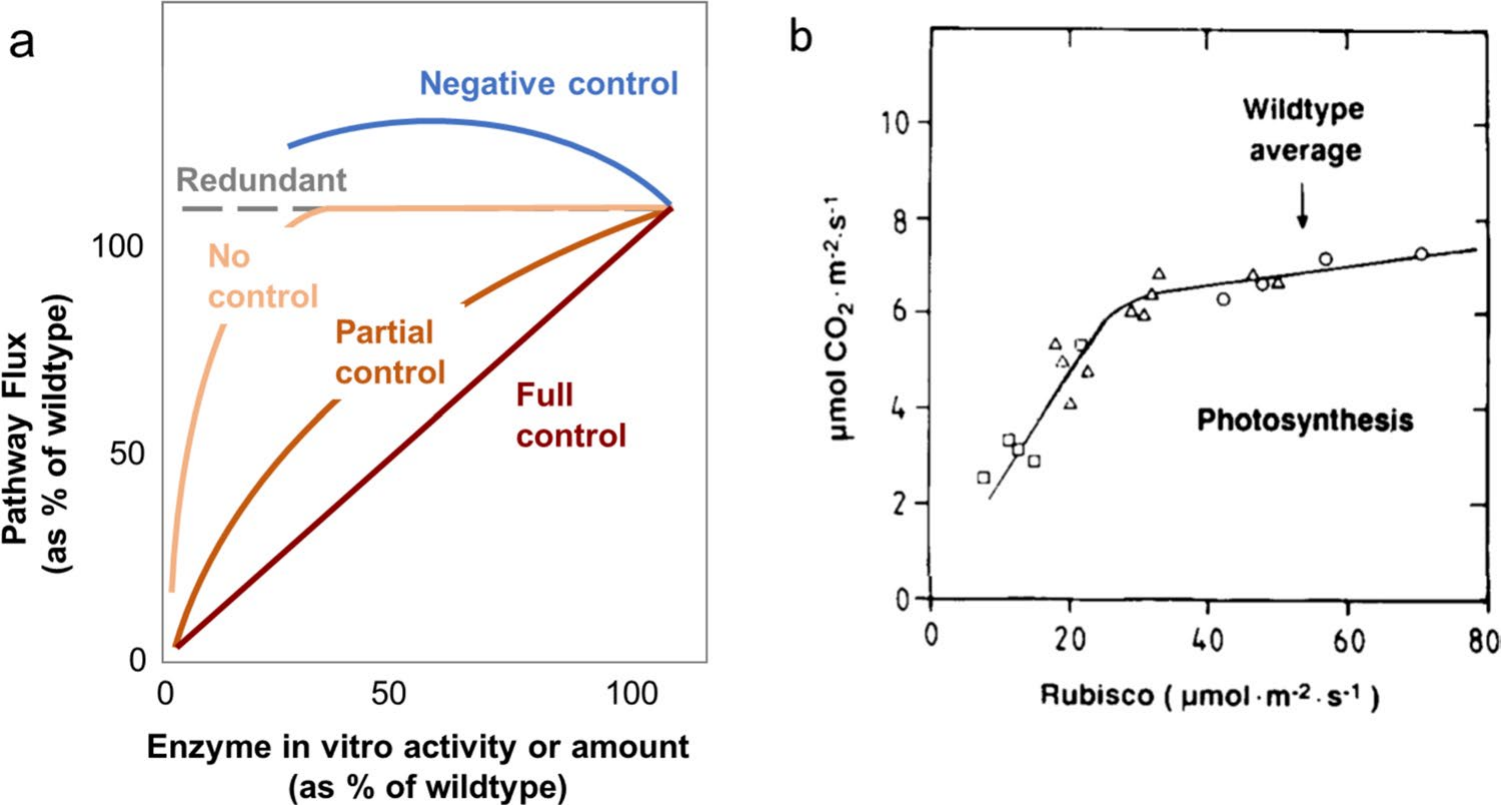
Explanation of the concept of the flux control coefficient (FCC) and an example from Quick et al. (1991a) of the experimental determination of the FCC of Rubisco using antisense *rbcS* lines. **a** FCC is estimated from a plot of pathway flux against enzyme amount or enzyme in vitro activity. Here, for simplicity, both axes are normed to the wild-type value and the FCC can be read off the plot as the initial slope when Rubisco is decreased slightly below the wild-type level. The mathematical formulation of FCC is based on infinitesimal changes but, in practice, a change of 20–30% or more is needed to obtain reliable experimental data for the change in enzyme abundance and the impact on pathway flux. If the initial slope = 1, the enzyme exerts full control (FCC = 1, deep red). If there is a slope but it is less than 1, the enzyme exerts partial control (dark orange): the FCC is equal to the initial slope. If there is no initial slope, the enzyme does not exert control (light ochre) (FCC = 0). In this case, flux is usually inhibited when a large part of the enzyme is removed. The case is also illustrated where even complete removal of the enzyme has no impact on pathway flux (gray dotted line, ‘redundant’). Finally, if there is a negative slope (i.e., flux increases when there is a small decrease in enzyme activity/amount below the wild-type value), the enzyme has a negative FCC (blue). **b** Example of the relation between Rubisco in vitro activity and the net rate of photosynthesis, modified from Quick et al. (1991a). The experiments were performed using wild-type tobacco (○), segregating progeny of F1 antisense *rbcS* line 3 (Δ) that contained no (i.e., pseudo-wild-type), 1 (giving a small decrease in Rubisco) or 2 copies (giving a larger decrease in Rubisco) of the antisense construct, and antisense *rbcS* line 5 (□) that had a large reduction in Rubisco activity. Rubisco activity was measured in optimized conditions after preincubation to fully activate Rubisco. Preliminary experiments showed that there was close agreement between measured in vitro Rubisco activity and the amount of Rubisco protein. Net CO_2_ fixation was measured by gas exchange in growth conditions (550 µmol m^−2^ s^−1^ irradiance, 350 ppm CO_2_, 20 °C). The scaling of the *y* and *x* axis was chosen, such that the average wild-type Rubisco activity and average wild-type net CO_2_ fixation are geometrically similar. When Rubisco is decreased by ~ 40% below the average wild-type value, net CO_2_ fixation is only weakly inhibited (slope of ~ 0.15). A strong proportionally does not appear until Rubisco is decreased to below half the average wild-type value. Similar data were obtained in many independent experiments in these conditions (e.g., Quick et al. 1991b, 1992; Lauerer et al. 1993: summarized in Fig. 3a)

A radically different approach was proposed by Kacser and Burns (1973). They proposed that regulation should be studied by decreasing the abundance of an enzyme and investigating the resulting change in pathway flux. The contribution of the enzyme to control could be inferred by comparing the magnitude of the decrease in enzyme abundance/activity with the magnitude of the decrease in pathway flux. Formally, a flux control coefficient, FCC, could be calculated as FCC = (dJ/J)/(dE/E) where dE/E is the fractional change in enzyme abundance and dJ/J is the fractional change in pathway flux. This is illustrated in Fig. 1a, where the axes are scaled, such that FCC is given by the initial slope, i.e., the response to a small decrease of enzyme abundance below that in the wild type. If the initial slope is I, the enzyme controls pathway flux. If there is a tangible but weaker initial slope, the enzyme contributes to control but is not the sole controlling enzyme. If the line is initially flat, the enzyme does not control flux. If a small decrease in enzyme abundance leads to an increase in flux, the enzyme has a negative FCC (this may sound theoretical but it does occur, for example at a branchpoint where decreasing abundance of an enzyme in one branch may increase flux into the other branch). Figure 1a also illustrates the very important point that the slope typically becomes stronger as more enzyme is removed. Indeed, if this were not the case then slope would be flat until no enzyme is left, i.e., the enzyme is either redundant or not involved in the pathway. This is why investigation of control is not possible using a large decrease in enzyme abundance—in this case, the data can only be used to show that the enzyme is required, but not to assess its contribution to control of the pathway.

At that time, I was studying in Cambridge, UK. In autumn 1975, I took a lecture series given by Tom ap Rees on metabolic regulation. Tom gave a lucid presentation of the ‘Newsholme and Start’ approach. However, in his last lecture, he came into the lecture room looking slightly agitated. He started by saying he was wondering if everything he had said up till then might be wrong, and walked us through the ideas of Kacser and Burns. Tom ended with a conclusion along the lines ‘*I think they are probably correct, but I don’t think it can be done’*. Where were the mutants for all the individual enzymes in all of the pathways? And in the few cases where mutants were available, they typically involved large changes. And there was no way to generate mutants at will, and certainly not in plants. An alternative approach would be to use inhibitors, but to quantify how much enzyme had been titrated out would need tight-binding inhibitors; these were rare and there would still be the lurking risk of non-specificity.

That is where things stayed, at least for me, for the next 12 or so years as I carried out my PhD with Tom, worked in the lab of Hans Heldt in Munich and Göttingen, and then set up a lab in the department of plant physiology in Bayreuth. However, in the intervening time, EMS mutagenesis and screens in Arabidopsis were starting to generate mutants, for example in Chris Somerville’s lab by screening for low starch to identify mutants in starch biosynthesis (for references see Stitt et al. 2010). I wondered if we could learn anything about the control of starch synthesis by creating heterozygotes where we had a 50% decrease in enzyme abundance. Also, determined evolutionary biologists like Leslie Gottlieb in Davis were creating mutants to study the fitness advantage of duplicated genes like cytosolic phosphoglucoisomerase in *Clarkia xantiana*, providing an even more graded decrease in enzyme abundance. Our early studies in Bayreuth (Neuhaus et al. 1989; Neuhaus and Stitt 1990) showed that the approach of Kacser and Burns was feasible. For example, we saw that ADP glucose pyrophosphorylase exerts partial control over starch synthesis. We also saw things we had not expected; that plastid phosphoglucomutase exerted partial control of starch synthesis in high irradiance, and that cytosolic phosphoglucoisomerase exerted partial control over sucrose synthesis in both low and high irradiance, despite these 2 enzymes catalyzing near-equilibrium reactions. Further, we saw that control could be shared between enzymes and that the distribution of control depended on the conditions. Even more excitingly, our multilayered analytical platforms allowed us to measure the response of other metabolites and signal metabolites like fructose 2,6-bisphosphate, giving insights into the impact on the rest of the pathway and revealing why pathway flux was (or was not) inhibited. Like dropping a stone and watching the ripples spread through the pond.

## The advent of plant transformation

However, the big problem remained. There were still too few mutants, and it was almost impossible to obtain mutants with a graded decrease in expression of an enzyme that, for some reason, you really wanted to look at, let alone to do this for many enzymes from a pathway. This changed with the advent of *Agrobacterium*-mediated plant transformation and, by a quirk, the inability to generate gene knock-outs by homozygous recombination in plants. This led to adoption of ‘antisense’ technology, in which an open-reading frame was expressed in the reverse (‘antisense’) orientation to create a mirror-copy that bound the endogenous mRNA and triggered its degradation. This approach typically generated many lines that varied in the extent to which the abundance of the targeted protein was decreased. Rodermel et al. (1988) were among the first to use antisense technology to address a biological question; they wanted to know if the abundance of Rubisco depends on expression of the nuclear-encoded small subunit of Rubisco (RBCS), or the plastid-encoded large subunit (RBCL). To do this, they generated a set of antisense *rbcS* lines in tobacco in which the RBCS protein was decreased to a varying extent. This sufficed to decrease total Rubisco abundance, with the excess RBCL protein being degraded.

## Rubisco as a very important enzyme in photosynthesis

Paul Quick, Ekkie Neuhaus and myself read Rodermel et al. (1988) with great excitement, not just because of the technology but also because of the enzyme that had been targeted. In the preceding years, building on a ground-breaking mechanistic model from the ANU group in Canberra (e.g., Farquhar et al. 1982), Rubisco had emerged as a key regulatory protein of photosynthesis (Woodrow and Berry 1988). The model predicted that photosynthesis was limited by Rubisco in saturating light, and by the regeneration of ribulose 1,5-bisphosphate (RuBP) in limiting light. This followed partly from the low k_cat_ of Rubisco, making it necessary for the leaf to invest a substantial amount of its protein in this single enzyme. It also followed from the trade-off between k_cat_ and the specificity for CO_2_ compared to the competitive reaction with O_2_, meaning that under atmospheric CO_2_ concentrations Rubisco would be limited by CO_2_. The model was already a driving force in basic photosynthesis research, and since then, it has played a key role in global carbon cycle models. The antisense lines in Rodermel et al. (1988) looked to be the perfect genetic material to test these ideas experimentally, especially as some of the genotypes had a relatively small (30–40%) reduction in Rubisco abundance. Paul and Ekkie suggested we write (literally, no email back then!) to Steve Rodermel and Laurie Bogorad at Harvard and ask if they would send us some seeds. Out of the blue, about 3 weeks later, a large box of seeds arrived in the post. Enough to do a lot of experiments.

Luckily, thanks to a cooperation we had initiated with Lothar Willmitzer, Uwe Sonnewald, and Antje von Schaewen, we had just established the formalities to work with transgenic plants (there were no laws at that time, but scientists voluntarily registered that they were using gene manipulated organisms). A vital enabler for the experiments that followed was an on-going cooperation with Detlef Schulze from the neighboring Department of Plant Ecology, in which we were comparing photosynthesis and growth in different wild species. Up till then, we had been basically a plant biochemistry lab, performing our measurements of photosynthesis with wildly over-saturating CO_2_ concentrations in David Walkers leaf disc O_2_ electrode. This was obviously not the way to study how decreased Rubisco abundance affected photosynthesis. I remember going to Detlef and saying something like ‘*Detlef, we’ve been trying to understand the relationship between photosynthesis and growth by comparing species, but there are so many other things changing that we can hardly interpret the results. I’ve got some transgenic tobacco plants where photosynthesis is decreased by them having less Rubisco, without anything else being changed. I think this is a much better experimental system*’. Detlef immediately answered something like ‘*Let’s go for it!!*’. This gave us access to his state-of-the-art plant growth chambers and gas exchange facilities, and allowed us to work alongside young scientists who were experts in their use, like Uli Schurr, Klaus Fichtner, and Marianne Lauerer. Without this, the following experiments would have been impossible.

## Impact of a small decrease in Rubisco abundance

**Fig. 2:**
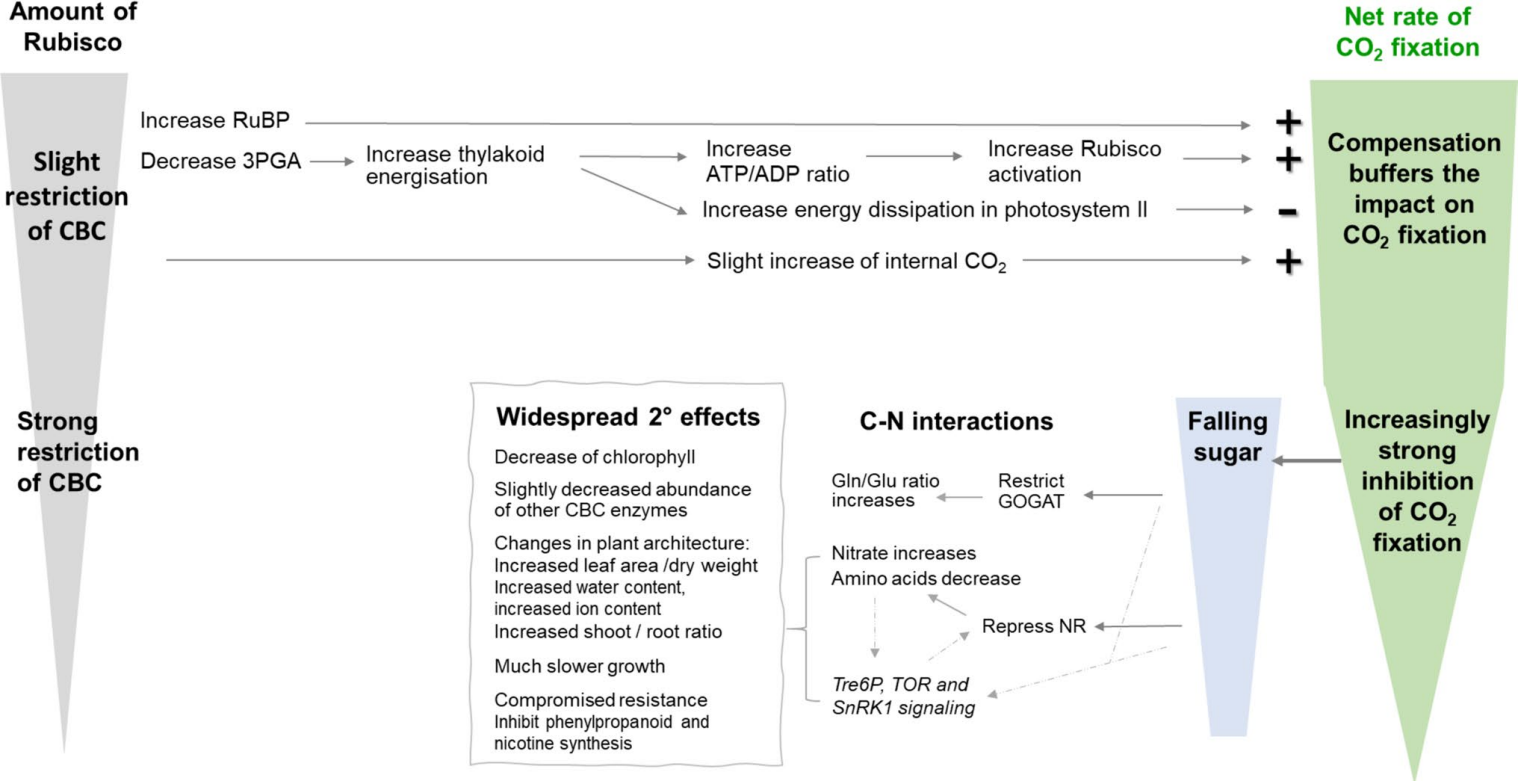
Direct and indirect consequences of a decrease in Rubisco abundance. This analysis is based on analyses performed on plants grown at 550 µmol m^−2^ s^−1^ irradiance, 350 ppm CO_2_, 20 °C, and with photosynthesis measured in growth conditions. The upper part of the scheme summarizes the immediate direct response to a moderate ($\sim$40%) decrease in Rubisco abundance. There is an increase of the substrate (RuBP) and decrease of the product (3PGA). The decrease in 3PGA leads to a slight decrease in demand for ATP and NADPH from the thylakoid light reactions, resulting in an increase of the ATP/ADP ratio. This activates Rubisco activase (Portis et al. 2008), leading to an increase in the proportion of total Rubisco that is carbamylated and catalytically active. There is also a slight increase in stomatal conductance leading to higher internal CO_2_ concentration in the leaf. At the same time, the increase in thylakoid energization activates energy dissipation at photosystem II. Modeling indicated that in genotypes with an average 43% reduction in Rubisco abundance, the increase in RuBP, Rubisco activation, and CO_2_ concentration would lead to a compensating 3.5, 31, and 1.1%, respectively, gain in photosynthesis, largely compensating for the lower Rubisco abundance. The increase in energy dissipation was modeled to lead to a 6.2% decrease in the rate of photosynthesis, which is only slightly smaller than the measured decrease in photosynthesis (6.6%) (Quick et al. 1991a). Thus, in these conditions of slightly limiting irradiance, a moderate decrease in Rubisco abundance is largely buffered by compensation that allows the remaining Rubisco to operate more efficiently, and there is only a small inhibition of photosynthesis due to side-effects on energy dissipation. There is also a small decrease in water use efficiency due to increased water loss per fixed CO_2_. The lower part of the scheme summarizes the response when Rubisco abundance falls below about 50%. Photosynthesis is inhibited more strongly, and much more widespread effects appear, of which many are probably indirect. Inhibition of photosynthesis leads to a decline in sugar levels (Quick et al. 1991b; Fichtner et al. 1993). Probably as a consequence, there is an inhibition of ammonium assimilation in the GOGAT pathway. This is indicated by an increase in the glutamine:glutamate (Gln:Glu) ratio, which has been seen in many studies of C-limited plants due to inhibition of flux over PEP carboxylase and a decline in 2-oxoglutarate levels (2-oxoglutarate was not measured in the studies of antisense *rbcS* lines). When sugars fall further, nitrate reductase (NR) is repressed and is also post-translationally inactivated (Vincentz et al. 1993; for further references, see Heldt and Piechulla 2021). This explains the accumulation of nitrate and a general decline of amino acids in antisense lines with strongly decreased Rubisco abundance (Quick et al. 1991b, 1992; Stitt and Schulze 1994; Matt et al. 2002; Fritz et al. 2006). This combination of falling sugars, rising nitrate, and falling amino acids in turn triggers a plethora of secondary responses including a large decrease of chlorophyll, possibly also in part from photodamage due to pressure in photosystem II (Quick et al. 1991a, b, 1992; Fichtner et al 1993; Lauerer et al. 1993) and, once Rubisco fell below about 25% of wild-type levels, decreased abundance of some other Calvin Benson cycle enzymes (Quick et al. 1991a). There was a collapse of the levels of secondary metabolites involved in defense including phenylpropanoids and nicotine (Matt et al. 2002). Subsequent work has found similar changes in response to sugar depletion and is investigating the molecular mechanisms, which include trehalose 6-phosphate, SnRK1, and TOR-signaling. There was an increase in leaf area associated with increased water and ion content (Quick et al. 1991b; Fichtner et al. 1993; Lauerer et al. 1993), an increase in the shoot/root ratio (Fichtner et al. 1993), and slower growth (Quick et al. 1991b; Fichtner et al. 1993; Lauerer et al. 1993). These changes in plant architecture may, at the whole plant level, partly compensate for the falling rate of photosynthesis by increasing leaf area and light interception per invested dry weight. However, other changes, including the decline of chlorophyll and other Calvin–Benson cycle enzymes, will exacerbate the decline of photosynthesis

In the first set of experiments (Quick et al. 1991a), we grew the antisense lines at moderately high irradiance (550 µmol m^−2^ s^−1^) with excess nitrogen fertilizer. In the different genotypes, Rubisco was decreased about 35–40, 60, and 90% below the average wild-type content. There was only a $\sim$7% decrease in photosynthesis when Rubisco was decreased by 35–40%, allowing an FCC of about 0.15 to be estimated, and a steep decline when Rubisco was decreased further (for details, see legend to Fig. 1b). We then used our multilayered analytic platform to investigate how photosynthesis responds when Rubisco is decreased by 35–40%—what is buffering photosynthesis? Details are given in the upper part of Fig. 2. Briefly, the decrease in Rubisco abundance was largely compensated by an increase in post-translational activation (carbamylation) of Rubisco and an increased level of the substrate ribulose 1,5-bisphospate (RuBP). Based on later research, the increase in carbamylation was probably due to an increased ATP/ADP ratio promoting reductive activation of Rubisco activase (see Portis et al. 2008). The downside was that the higher ATP/ADP ratio was accompanied by an increase in thylakoid membrane energization, which led to increased energy dissipation in photosystem II. This was the main proximal reason for the small inhibition of photosynthesis.

## Short-term conditions and prehistory affect the extent to which Rubisco controls photosynthetic rate

**Fig. 3:**
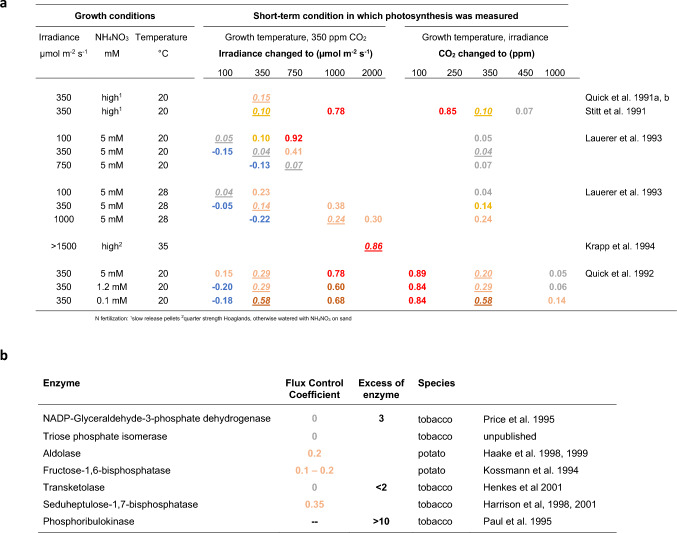
Flux control coefficients in the Calvin–Benson cycle. The value of the flux control coefficient is colored following the scheme of Fig. 1: red = FCC approaching 1; dark to light orange = partial control; gray = no control; blue = negative FCC. **a** Flux control coefficients of Rubisco measured in *rbcS* antisense tobacco plants grown in different conditions (irradiance, temperate, nitrogen fertilization) and measured in different short-term conditions (different irradiance, different CO_2_ concentration). Measuring conditions for photosynthesis that correspond to the growth condition are indicated by italics and underlining (e.g., *0.2*). The FCC in growth conditions was moderate, except for plants grown at high temperature and very high irradiance, when the flux control coefficient was high (0.86, Krapp et al. 1994) and plant grown in low nitrogen (0.58, Quick et al. 1992). When photosynthesis was measured in other conditions than those used for growth, this represents a short-term change in conditions, as the plant were only exposed to the measuring condition for a short time before starting the measurements. The FCC increased when photosynthesis was measured at higher irradiance than growth irradiance and decreased when photosynthesis was measured at a lower irradiance than growth irradiance. The FCC increased when photosynthesis was measured at a lower CO_2_ concentration and decreased when photosynthesis was measured at a higher CO_2_ concentration than ambient CO_2_ (350 ppm). Plants grown on low NO_3_NH_4_ had higher FCCs than plants grown in nitrogen-replete conditions. When net CO_2_ fixation was measured at low irradiance in high irradiance-grown plants, negative flux control coefficients were found, i.e., genotypes with a moderate reduction in Rubisco had higher rates of net CO_2_ fixation than wild-type plants (blue font). **b** Summary of collated FCCs of other Calvin–Benson cycle enzymes measured in growth conditions (for details, see Kossmann et al. 1994; Price et al. 1995; Paul et al. 1995, 2000; Haake et al. 1998, 1999; Harrison et al. 1998; Henkes et al. 2001). ‘Excess of enzyme’ indicates the fold decrease in enzyme abundance until net CO_2_ fixation started to decline. Rubisco is not the only Calvin–Benson cycle enzyme with a tangible flux control coefficient. Notably, 2 enzymes that catalase readily reversible reactions had detectable control coefficients (aldolase) or started to restrict photosynthesis when their abundance was decreased by less than 50% (transketolase)

One important prediction of the Farquhar, von Caemmerer, and Berry model (1982) was that the contribution of Rubisco to control of photosynthesis would depend on the conditions. The *rbcS* antisense lines allowed us to test this by varying the irradiance and CO_2_ concentration under which photosynthesis was measured and, in further experiments, by varying the conditions in which the plants were grown (Fig. 3a).

Briefly, we consistently found that the FCC of Rubisco increased when photosynthesis was measured in higher irradiance and decreased when photosynthesis was measured in lower irradiance, and that the FCC increased when photosynthesis was measured in lower CO_2_ and decreased when photosynthesis was measured in higher CO_2_ (Stitt et al 1991). This qualitatively matched the predictions of the Farquhar, von Caemmerer, and Berry model. Further, these responses to current irradiance and CO_2_ concentration were robust; they were found in plants that had been grown in different irradiance (Lauerer et al. 1993), temperature (Lauerer et al. 1993), and nitrogen fertilization (Quick et al. 1992) regimes.

We also found that the FCC depended on the prehistory of the plants (i.e., here, the growth conditions). This is because the growth condition can alter the balance between Rubisco abundance and other components of the photosynthetic apparatus. The FCC measured at a given irradiance and CO_2_ concentration was higher, and often much higher, in plants that had been grown in low irradiance compared to plants that had been grown in high irradiance. The FCC also increased markedly when plants were grown on a low nitrogen supply (Quick et al. 1992). Under limiting nitrogen, Rubisco abundance decreased and became quite strongly limiting for photosynthesis in ambient conditions (FCC $\sim$0.58) whereas under high nitrogen supply, there was typically a small excess of Rubisco over that which was strongly limiting for photosynthesis. This small excess allowed an increased rate of photosynthesis in response to a short-term increase in irradiance. Changes in the relation between Rubisco abundance and the remainder of the photosynthetic apparatus had been described in earlier studies of wild-type plants growing in different conditions, and it was satisfying to see that these changes really did modify the functional balance between Rubisco and the rest of the photosynthetic machinery.

There was one apparent discrepancy between our experimental determinations of the FCC of Rubisco, and the predictions of the Farquhar, von Caemmerer, and Berry model. Whereas the model predicted that Rubisco would strongly limit photosynthesis in high irradiance, in many of our treatments, Rubisco was only partly limiting. This was partly because many of our early photosynthesis measurements were carried out in moderately limiting irradiance (see also Hudson et al. 1992, and Andrews et al. 1996 for discussion). We obtained very high FCCs when plants grown in low or moderate irradiance were analyzed in high irradiance (FCC $\sim$0.92 and 0.78, respectively). An extremely high FCC ($\sim$0.86) was also obtained when plants were grown in a greenhouse in Lisbon under very high light and photosynthesis was measured in these conditions (Krapp et al. 1994). That we never saw an FCC of 1 may be due to experimental noise or to heterogeneity in the leaf, including shading in the lower part of the leaf.

We sometimes observed a negative FCC, i.e., lines with less Rubisco had a higher rate of photosynthesis. This was seen after shifting high-light grown plants to low irradiance (Lauerer et al. 1993), especially when they were grown on low nitrogen (Quick et al. 1992). It probably reflected increased investment in chlorophyll or light harvesting components, using nitrogen made available by the genetically forced decrease of Rubisco protein. These observations were intriguing; they implied that the balance between Rubisco abundance and the apparatus for light-capture in high irradiance-grown wild-type plants is sub-optimal for photosynthesis in low irradiance. Nowadays, there is great interest in photosynthetic performance in the field in fluctuating environments (Kaiser et al. 2014). Our old studies with antisense *rbcS* lines highlight that there will be trade-offs, and illustrate how these trade-offs can be investigated using appropriate genetic material.

## Other Calvin–Benson cycle enzymes also exert control

The finding that the experimentally determined FCC was always somewhat below 1 and often much lower indicated other factors co-limit photosynthesis. In the 1990 s, we and others used antisense lines in tobacco and other species to investigate the contribution of other Calvin–Benson cycle (CBC) enzymes that are involved in the reduction of 3PGA and regeneration of RuBP to the control of photosynthetic rate (Fig. 3b). These studies earmarked plastid fructose-1,6-bisphosphatase and especially seduheptulose-1,7-bisphosphatase as co-limiting for photosynthesis in ambient growth conditions. Subsequent research by Christine Raines and colleagues showed that, in some species, photosynthetic rate could be enhanced by overexpressing seduheptulose-1,7-bisphosphatase. This has been highlighted as one strategy to increase photosynthesis in the field (see Raines 2011; Long et al. 2025).

Phosphoribulokinase catalyzes a highly irreversible reaction and is known to be a highly regulated, in the sense of being subject to feedback regulation by multiple allosteric effectors (see Heldt and Piechulla 2021). It nevertheless did not exert any control over photosynthetic rate in ambient growth conditions (Fig. 3b). Later, an elegant study by Paul et al. (2000) showed that phosphoribulokinase did exert some control (FCC $\sim$ 0.25) when plants were grown in low irradiance and suddenly shifted to high irradiance, and that this was due to the relative abundance of CBC enzymes differing between low and high-irradiance-grown plants. This is reminiscent of the response of the FCC of Rubisco to growth and short-term irradiance. It is likely that further enzymes show similar imbalances after sudden changes in conditions and that this will affect performance in fluctuating environments.

Studies in the 1990 s also revealed that aldolase has a tangible FCC in a range of growth conditions (Haake et al. 1998, 1999) and that transketolase is only in small excess above the abundance at which it starts to limit photosynthesis (Henkes et al. 2001) (Fig. 3b). This was initially unexpected, because these enzymes (see also above, phosphoglucomutase and phosphoglucoisomerase) catalase near-equilibrium reactions and it was widely assumed that these kinds of enzyme were in large excess (see first section). These observations led us to realize that the crucial issue is not whether an enzyme catalyzes a reversible or irreversible reaction but, instead, its connectivity with the rest of the pathway. Phrasing the idea in current terms, it depends on network topology. The immediate response to a decrease in the abundance an enzyme is an increase in the level of its substrate and/or a decrease in that of its product and, where relevant, an increase in the level of positive effectors and/or a decrease in the levels of negative effectors. This allows the remaining enzyme to be used more efficiently. Viewing the pathway as a network, these direct adjustments will not influence pathway flux until one or more of the adjustments starts to inhibit other enzymes in the pathway. Seen from this perspective, it is irrelevant if an enzyme catalyzes a reversible or an irreversible reaction. Indeed, there may be more flexibility when an enzyme has multiple regulatory effectors than when the only available response is to increase its substrate and/or decrease its product.

## Impact of slower photosynthesis on wider aspects of metabolism

Antisense *rbcS* lines also allowed us to investigate the impact of a lower rate of photosynthesis on the rest of metabolism and (see next section) whole plant allocation and growth. Although these questions had already been studied, the experimental approach usually involved changes in irradiance, which might influence not only photosynthetic rate but also many other processes due to altered light-signaling, or cross-species comparisons. Research using elevated CO_2_ to increase photosynthesis started in the 1980 s but was at that time often compromised by side-effects due to small pot size and falling nutrient availability.

Figure 2 (lower part) shows some of the metabolic responses that we observed when Rubisco abundance was decreased so far that it strongly inhibited photosynthesis. As expected, carbohydrate levels decreased. There were shifts in amino acid levels including an increase of the glutamine:glutamate ratio indicating that ammonium assimilation in the GOGAT pathway is restricted by low 2-oxoglutarate. When photosynthesis was decreased further, nitrate reductase activity fell strongly, nitrate accumulated and there was a general decrease of amino acids (Stitt and Schulze 1994; Fritz et al. 2006). Subsequent work showed that low sugar represses nitrate reductase (Vincentz et al. 1993; see also Heldt and Piechulla 2021). We also looked beyond central metabolism, prompted by a chance observation of Anne Krapp and colleagues in Lisbon. Some mice got into the greenhouse but they ate only the tobacco plants with the lowest Rubisco abundance, and there were no dead mice. Knowing the LD50 of nicotine for humans, the weight of a mouse, and the amount typically present in wild-type tobacco, we surmised that nicotine content must be strongly decreased in the very low-Rubisco mutants. This triggered a project in which we found that nicotine and also phenylpropanoid metabolites were indeed strongly reduced (Matt et al. 2002). This was one of the first studies linking maintenance of defense metabolites to photosynthetic performance. Altogether, the antisense *rbcS* lines allowed us to see the impact of a single intervention rippling out through metabolism. Back then, we were not talking about networks, let alone systems biology. The phenotypes of the antisense lines told us that is how we should be thinking, because that is how the biology is.

## Impact of a slower photosynthesis on allocation and growth

As mentioned above, one of our preceding projects was a comparative investigation of allocation, plant architecture, and growth in different species, together with the group of Detlef Schulze. The basic idea we were testing was whether high growth rates are linked to changes in architecture that maximize leaf area and light interception (Poorter 1990). Against this background, we asked whether decreased abundance of Rubisco impacted leaf composition and plant architecture. If so, we could investigate these whole plant traits in an otherwise identical genetic background and without complications due to use of different growth conditions.

Decreased Rubisco indeed led to increased leaf area per invested dry weight or per invested protein, due to the leaves being thinner, more brittle and having a higher water and ion content (Fig. 2; Quick et al. 1991b; Fichtner et al. 1993; Lauerer et al. 1993). Back then, we speculated that lower water use efficiency and accumulation of ions might drive the increase in leaf area. This was probably over-naïve, and it would be rewarding now to ask how low sugar restricts cell wall synthesis (Verbančič et al. 2018). We also observed a higher shoot–root ratio, which will increase leaf area per unit plant dry weight. It is well known that availability of nutrients like nitrogen and phosphate regulates the shoot/root ratio. It is less clear whether carbon supply has an analogous effect, and the role of sugar-signaling pathways like trehalose 6-phosphate, SNRK1, and TOR in regulating root growth is currently an important research topic (Morales-Herrera et al. 2024). Lines with decreased rates of photosynthesis due to genetic intervention, like the antisense *rbcS* lines, might be a useful resource for such studies. More generally, although first hints were emerging from work of Jen Sheen and our collaboration with Lothar Wilmitzers team that sugars regulate gene expression (Sheen 1990; von Schaewen et al. 1990), in 1991, the study of sugar-signaling pathways was in its infancy. The *rbcS* antisense lines were an appetizer, giving a first taste of the broad impact of sugar-signaling on plant metabolism and growth.

## Back to the future

Photosynthesis research has moved forward since the 1990 s, driven in part by genome sequencing, ‘omics analyses, and systems biology, gaining much deeper insights into molecular mechanisms, developing increasingly sophisticated models, and pursuing strategies to improve crop photosynthesis (see Long et al. 2025). However, approaches from the early 1990 s remain relevant. In particular, it is often important to use an appropriately graded genetic intervention rather than knock-outs.

It is now possible to deploy a spectrum of quantitative platforms to analyze metabolic and other phenotypes in a systems-oriented way, including ‘omics technologies developed in the last 25 years, and especially emerging platforms for monitoring post-translational regulation. This will allow more detailed insights into if, and how, targeted reduction in the abundance of an enzyme is compensated by altered transcription, turnover, and post-translational regulation of the targeted enzyme, as well as by analogous compensatory responses elsewhere in the metabolic network. Indeed, one short-coming of flux control analysis was that it assumed a stable reduction in abundance of the targeted enzyme would always be possible. This is not the case for enzymes that are subject to strong feedback regulation at the level of transcription, translation or protein stability (see e.g., Scheible et al. 1997 for an early case study with nitrate reductase). These are, however, often key enzymes for understanding how metabolic networks cope and adjust to different conditions.

Measurement of flux is at the core of the study of metabolic regulation. In the 1990 s, we just measured net CO_2_ assimilation. One of the major developments in photosynthesis research in the last 15 years was to combine ^13^CO_2_ labeling, mass spectrometry, and sophisticating modeling approaches to measure complex flux patterns in metabolic networks (see Koley et al. 2024). It would be fascinating to perform sophisticated measurements of fluxes on genetic material with progressive changes in the abundance of key components of the photosynthetic apparatus.

New technologies will allow important extensions of flux control analysis. Compared to antisense technology, better understanding of promotor function and gene editing will allow much more focused modification of expression, including cell-specific expression, of a gene-of-interest (see e.g., Jethva et al. 2024). This will allow dissection of control for enzymes and pathways that operate in between different tissues and cell types. Furthermore, a short-coming of flux control analysis in the last century was that it was restricted to altering enzyme abundance. Now, there is a much deeper understanding of protein structure and function, and gene editing will make it possible to carry out targeted changes in enzyme function, including altering the binding affinity for substrates and regulatory effectors, and altering the sites that allow post-translational regulation. This opens the possibility to assess the impact of graded changes in enzyme functionality on pathway operation.

An important shift in photosynthesis research has been an increasing emphasis on understanding performance in the field, especially under fluctuating conditions. Interactions between prehistory and short-term environmental changes is probably at the center of photosynthetic performance in the field. It would be fascinating to deploy plant material with graded change in enzyme abundance like the *rbcS* antisense lines (or graded changes in enzyme functionality) in fluctuating conditions to learn more about trade-offs and the underlying genetic determinants. Furthermore, one general message from the studies of the flux control is that in many growth conditions, there is some degree of co-limitation. This prompts the question: how in a fluctuating environment is information integrated to achieve this balance, how far is the balance achieved by regulating expression, and how important is post-transcriptional and post-translational regulation for maintaining a functional balance in fluctuating conditions?

Finally, the ability to measure an enormous number of parameters and the sheer multiplicity of scenarios that can occur in the field poses the challenge: how to integrate and use a huge amount of data. This will only be possible by modeling. However, it is unlikely that models that assume flux is optimized to meet a goal can be easily applied across a wide range of conditions, let alone fluctuating conditions, because the goal posts are always moving. Judicious use of plant lines with a partial reduction in the abundance of key enzymes or graded changes in their functionality will provide an invaluable tool to validate and parameterize realistic models of photosynthesis that work under field conditions.

## Data Availability

Not applicable.

## Abbreviations

FCC: Flux control coefficient
Rubisco: Ribulose-1,5-bisphosphate carboxylase/oxygenase
